# FtsK in motion reveals its mechanism for double-stranded DNA translocation

**DOI:** 10.1073/pnas.2001324117

**Published:** 2020-06-08

**Authors:** Nicolas L. Jean, Trevor J. Rutherford, Jan Löwe

**Affiliations:** ^a^Structural Studies Division, Medical Research Council Laboratory of Molecular Biology, Cambridge CB2 0QH, United Kingdom

**Keywords:** DNA translocation, chromosome segregation, bacterial cell division, cryo-EM

## Abstract

FtsK protein contains a fast DNA motor that is involved in bacterial chromosome dimer resolution. During cell division, FtsK translocates double-stranded DNA until both *dif* recombination sites are placed at mid cell for subsequent dimer resolution. Here, we solved the 3.6-Å resolution electron cryo-microscopy structure of the motor domain of FtsK while translocating on its DNA substrate. Each subunit of the homo-hexameric ring adopts a unique conformation and one of three nucleotide states. Two DNA-binding loops within four subunits form a pair of spiral staircases within the ring, interacting with the two DNA strands. This suggests that simultaneous conformational changes in all ATPase domains at each catalytic step generate movement through a mechanism related to filament treadmilling. While the ring is only rotating around the DNA slowly, it is instead the conformational states that rotate around the ring as the DNA substrate is pushed through.

## Significance

DNA motors are widespread molecular machines that hydrolyze ATP to generate movement. Among them, the bacterial protein FtsK is unusual in that it translocates on double-stranded DNA and is also the fastest known translocase. However, its translocation mechanism is poorly characterized, and there is currently no structural data of an active double-stranded DNA translocase bound to its substrate. We reveal FtsK’s mechanism to be related to hexameric helicases that move on single-stranded DNA, but uniquely adapted to the double-stranded substrate. Our work also highlights the concomitant conformational changes occurring all around the hexameric ring for each step of the reaction cycle.

DNA and RNA motors are a broad family of proteins that convert chemical energy from nucleotide triphosphate hydrolysis into motion relative to nucleic acids. Among them are ring-shaped helicases and translocases, which belong to the AAA+ and RecA families in eukaryotes and prokaryotes, respectively (1). With a translocation rate of up to 17.5 kb⋅s^−1^ (2), bacterial FtsK protein contains the fastest translocation activity known. FtsK is an essential part of the bacterial cell division machinery, recruiting downstream factors through its N-terminal transmembrane domain (3, 4). Its C-terminal cytoplasmic domain, which follows an often very long linker domain, can be subdivided into three modules: α, β, and γ, with β adopting the RecA-fold that contains the ATPase activity. It is the C-terminal αβγ-domain that is involved in chromosome segregation and dimer resolution (5–7). Upon recognition by the γ module of one direction-determining sequence in the genome, named KOPS (FtsK-orienting polar sequences) (8–12), α and β oligomerize as a homo-hexameric ring around double-stranded DNA (dsDNA) (forming FtsK_αβ_-dsDNA) (13). FtsK_αβ_ then starts pumping DNA, translocating toward the chromosome terminus where the γ-module activates recombination by recombinases such as XerCD (14, 15). Previously, FtsK_αβ_ had been crystallized as a sixfold symmetrical ring (13), but without dsDNA in the structure, the mechanism by which it translocates has remained unclear. Based on single-molecule experiments, structural data, and FtsK’s architecture, it was previously determined that FtsK_αβ_ loads upstream of KOPS sequences, resulting in DNA translocation from the β- toward the α-domains (10, 12, 13). Previous structures of DNA- or RNA-helicase complexes have suggested that nucleic acids, similarly to peptidic substrate in unfoldases, are generally recognized by a more-or-less asymmetrical ring through motifs organized into a partial helix, akin to a spiral staircase (16–22). However, the proposed translocation mechanisms for helicases are based on single-stranded nucleic acids, and it remains to be seen if similar mechanisms apply to motors translocating on more rigid double-stranded DNA substrates. To this end, we solved by electron cryo-microscopy (cryo-EM) structures of FtsK_αβ_ from *Pseudomonas aeruginosa* bound to dsDNA. A structure of FtsK_αβ_ translocating on DNA suggests that each catalytic step of the homo-hexamer is correlated with a concerted conformational change in all subunits, with each protomer in a different conformation.

## Results and Discussion

### Reconstitutions of FtsK_αβ_-DNA Complexes.

**Fig. 1.**
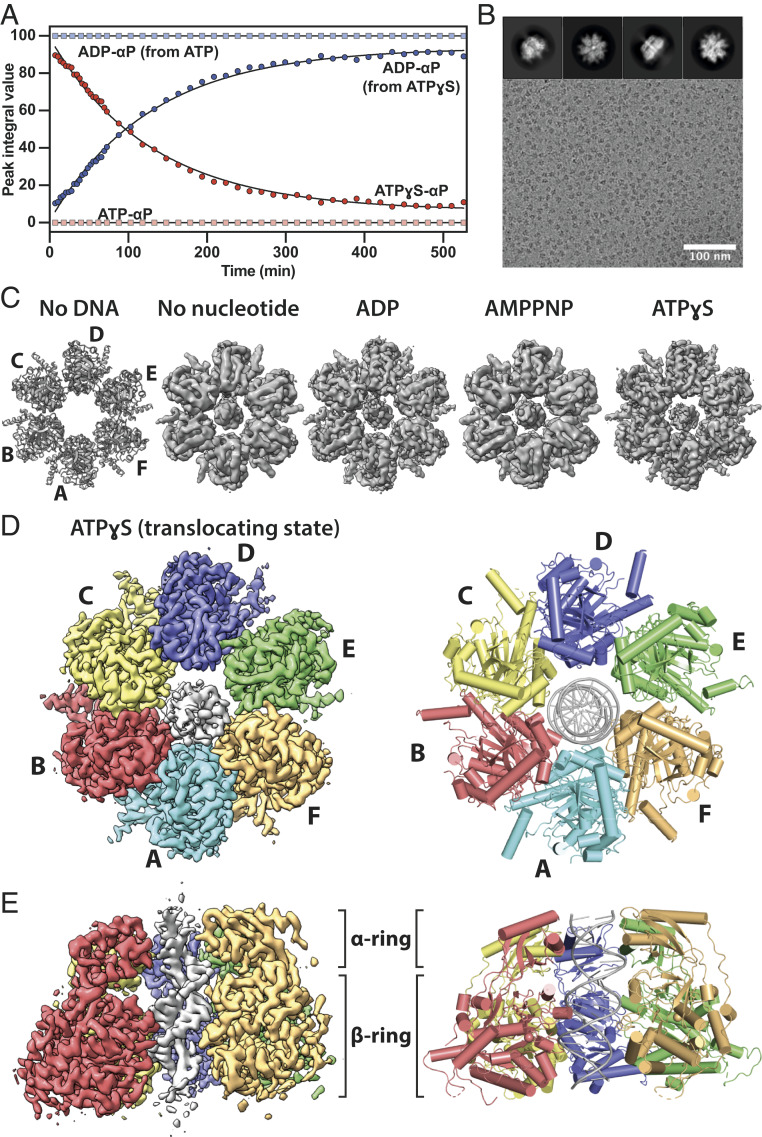
Cryo-EM structures of the FtsK_αβ_-DNA complex. (*A*) ATP and ATPγS hydrolysis by FtsK_αβ_ over time. The data points for the α-phosphates of ATP (light red), ATPγS (dark red), and ADP (light and dark blue) were determined from their peak integrals obtained by 1D ^31^P NMR experiments. ATP is fully hydrolyzed into ADP before the first data point, 7 min after nucleotide addition. (*B*) Representative cryo-EM micrograph of FtsK_αβ_-dsDNA + ATPγS. Typical 2D classes (*Top*). (*C*) Top views of previous FtsK_αβ_ crystal structure (PDB ID 2IUU) and FtsK_αβ_-dsDNA cryo-EM maps determined here. Only map III.E (translocating state, *SI Appendix*, Fig. S3) is shown for ATPγS. (*D* and *E*) A 3.6-Å resolution cryo-EM map (*Left*) of the FtsK_αβ_-dsDNA + ATPγS complex and refined atomic model (*Right*). Each subunit is colored differently with the double-stranded DNA in the pore. Shown as a top view, from above the α-ring through which the DNA exits the pore during translocation (*D*), and as a side view with subunit A removed (*E*).

Purified FtsK_αβ_ mixed with 45-bp dsDNA displays ATPase activity that is too fast for EM grid preparation and 1D ^31^P NMR (Fig. 1*A* and *SI Appendix*, Fig. S1), as expected from its previously determined turnover rate of 2,600 ATP s^−1^ per hexamer (23). In contrast, FtsK_αβ_ hydrolyzes ATPγS (but not AMPPNP) at a much lower rate (at least three orders of magnitude lower) (Fig. 1*A* and *SI Appendix*, Fig. S1). Although ATPγS cannot be a perfect mimic of ATP, we reasoned that this analog could help us observe more slowly but actively translocating FtsK hexamers. Cryo-EM with DNA, in the absence or presence of the nucleotides ADP, AMPPNP, and ATPγS, confirmed that FtsK_αβ_ forms homo-hexameric rings without the γ-module (Fig. 1*B* and *SI Appendix*, Fig. S2*A*), as observed previously (13). Although not easily visible in micrographs directly due to its small size, the 45-bp dsDNA can be detected inside the central pore after two-dimensional (2D) classification (Fig. 1*B*).

### Cryo-EM of Nontranslocating FtsK_αβ_-DNA Complexes.

Cryo-EM maps of the nucleotide-free, ADP- and AMPPNP-bound FtsK_αβ_-dsDNA complexes were obtained at resolutions of 4.91, 4.63, and 4.80 Å, respectively (Fig. 1*C* and *SI Appendix*, Table S1). In these nontranslocating states, the FtsK_αβ_ rings remained more-or-less symmetrical, with uniform nucleotide states around the ring, and no conformational differences relative to the previous crystallographic structure being obvious (Fig. 1*C* and *SI Appendix*, Fig. S2). Following from that, the DNA’s almost central position in the pore, in conjunction with the almost perfect αβ-ring symmetry, led to poor alignments around the DNA axis, and thus poor map quality of the DNA, since DNA does not have sixfold symmetry (*Materials and Methods*). In the symmetrical nucleotide-free, ADP- and AMPPNP-bound structures, most contacts with the dsDNA seem to occur at the N-terminal α-ring and the base of the β-subdomain where DNA enters the pore.

### Structure of FtsK_αβ_ Translocating on dsDNA.

The FtsK_αβ_-dsDNA-ATPγS sample was much more heterogeneous, the particles less symmetrical, and the DNA generally much better defined after three-dimensional (3D) refinement. Overall, three main particle classes could be distinguished, II.A, III.B, and III.E, each accounting for 4.9, 4.3, and 5.2%, respectively, of total particles selected after 2D classification and pruning for highest quality (*SI Appendix*, Fig. S3). The proportions of each of these classes increased to 30.0 (all II classes), 17.9 (III.B, III.D, IV.B, and IV.E classes), and 25.4% (III.A, III.C, III.E, IV.C, and IV.D classes), respectively, upon addition of particles from very similar classes that initially had lower resolutions and/or less defined DNA grooves. States II.A and III.B were reconstructed to 3.99 and 4.34 Å resolution and are in very similar conformations (*SI Appendix*, Figs. S4 and S5 and Table S2). Both structures are moderately asymmetric, mostly due to a movement of the β-subdomains in subunits A and B, turned away from the α-ring (*SI Appendix*, Fig. S5). Other subunits are again very similar to the previous crystallographic structure (13). The main difference between these two states lies in the length of the resolved dsDNA, which is 5 bp shorter in II.A on the side closest to the β-subdomain at the pore entry. From the maps we determined that in these structures ADP occupies all nucleotide-binding pockets, which suggests that they represent stalled states, with II.A having reached the end of the short 45-bp DNA after translocation.

Particles in the final class, III.E, were reconstructed into a map at 3.65-Å resolution (*SI Appendix*, Fig. S6 and Table S2) and result in a highly asymmetric ring with mixed nucleotide occupancy that is also slightly tilted relative to the DNA’s longitudinal axis (Fig. 1 *D* and *E* and *SI Appendix*, Fig. S4). For reasons explained below, we propose that class III.E represents the protein while it is actively translocating on the DNA, and from here on refer to it as FtsK_αβ_.

### Subunit Conformations within the Hexamer.

**Fig. 2.**
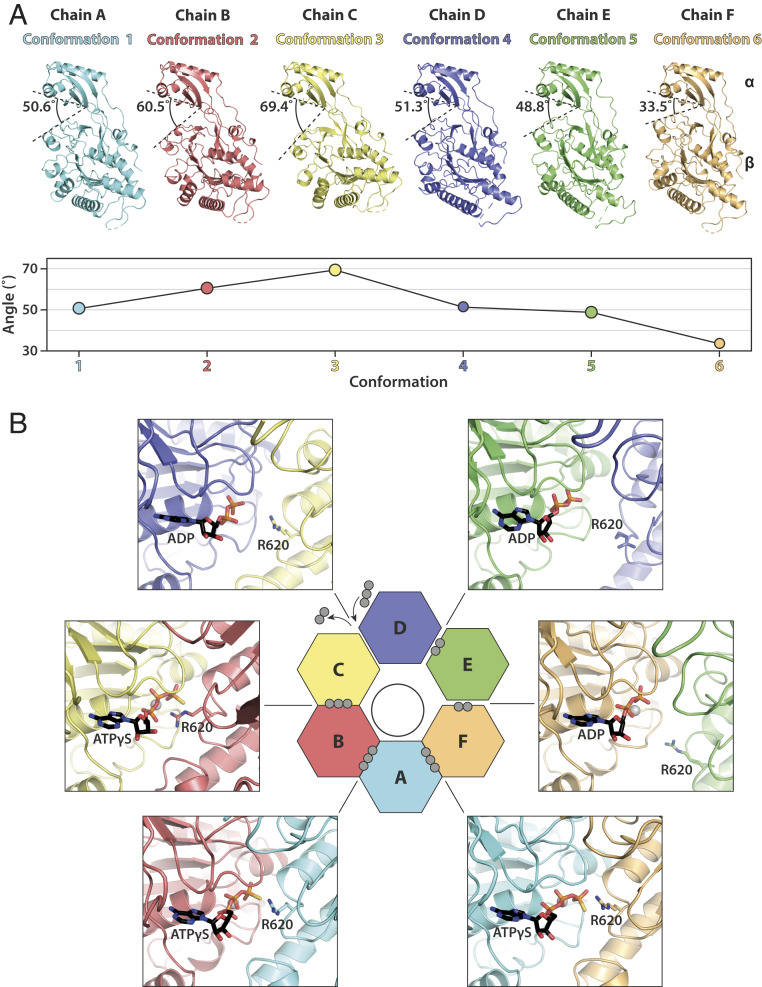
In the translocating state, FtsK_αβ_ subunits around the ring undergo a continuous conformational transition. (*A*) Side-by-side comparison of FtsK_αβ_ subunits aligned on the α-subdomains. Each subunit is in a different conformation, from here on identified by its color. The angle between α- and β-subdomains (between S383, I365, and K657 Cα) is indicated and plotted in the graph below to highlight the approximate wave nature of the intersubdomain angles. (*B*) Nucleotide states around the FtsK_αβ_ ring of the ATPase sites that are located at the interfaces between two neighboring subunits. Although ADP is modeled in the C-D pocket, inspection of the EM map suggests that it is likely in exchange with ATPγS. The activity-controlling arginine finger (R620) is highlighted. Gray spheres indicate phosphates per nucleotide.

In FtsK_αβ_ solved under ATPγS turnover conditions, each of the subunits A to F adopts a different conformation, named here 1 to 6 (Fig. 2*A*). The orientations and positions of the β-subdomains relative to the α-subdomains vary around the ring, describing mostly a planar rocking motion approximated by the angle between the S383-I365-K657 Cα atoms. The subdomains are closest to each other in conformation 6, with a measured angle of 33.5°, similar to the crystallographic structure. Going clockwise around the ring (when looking from the top where the α-subdomains are, as in Fig. 1*D*), β moves away from α and also toward the adjacent subunit on its left. A maximum is reached in conformation 3 with an angle of 69.4° after a gradual increase through conformations 1 and 2. The ATPase subdomain finally resets to conformation 6 through conformations 4 and 5. Therefore, the intersubdomain angles describe roughly a conformational wave (Fig. 2*A*, *Bottom*). The conformational diversity within the subunits of the FtsK_αβ_ hexamer results in an asymmetrical β-ring, which also creates a significant variation in intersubunit packing, as highlighted by the 65% decrease in buried subunit interface area between interfaces 2-3 and 5-6 (*SI Appendix*, Fig. S7). The subunit packing in conformations 5 and 6 might be loose enough to enable some freedom in β-movement without compromising the integrity of the ring. The lower resolutions reported for these two states support this hypothesis. In contrast, rotational symmetry in the α-ring is mostly preserved, giving the impression of a rigid aperture (*SI Appendix*, Fig. S7).

### Nucleotide States Around the Ring.

The conformational diversity within the FtsK_αβ_ ring is correlated with three different nucleotide states around the β-ring (Fig. 2*B* and *SI Appendix*, Fig. S8). In RecA-like hexameric translocases, active-site pockets are located at the subunit interfaces, near the outside of the rings. In FtsK_αβ_, ATPγS is well resolved at the F-A, A-B, and B-C interfaces (between conformations 6 and 1, 1 and 2, and 2 and 3). In the ATPγS-bound state, the subunit packing allows the arginine finger (R620) from one subunit to reach the nucleotide in the pocket of its adjacent subunit. Two other interfaces, D-E and E-F (between conformations 4 and 5, and 5 and 6), clearly show map densities for only two phosphates of ADP molecules, generated through in situ ATPγS hydrolysis. In agreement with a posthydrolysis state, here the arginine finger is located away from the nucleotide pockets. However, the two interfaces show subtle differences, such as an Mg^2+^ ion coordinated only in the E-F pocket and a much higher atomic B-factor for the ADP in the E-D pocket (90.1 versus 58.7 Å^2^). Finally, the nucleotide pocket at the C-D interface (between conformations 3 and 4) shows less well-defined nucleotide density for the two ADP phosphates. An additional density for a γ-phosphate, visible only at lower thresholds, suggests that ADP and fresh ATPγS are likely in exchange in this pocket. In agreement with such a transitional state, the arginine finger lies in a position midway between the ATPγS and ADP states.

### Interaction with the DNA Substrate.

**Fig. 3.**
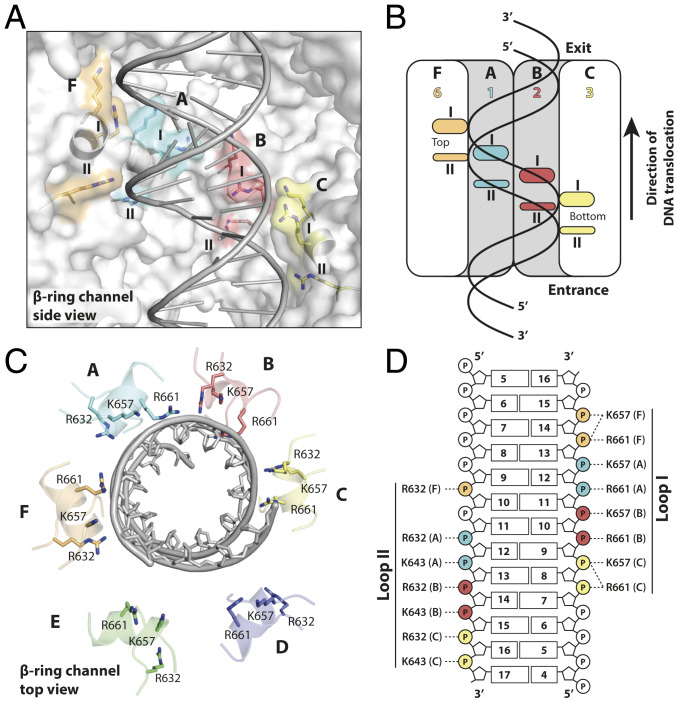
Interactions with double-stranded DNA within FtsK’s translocating pore. (*A*) Recognition by DNA-binding loops organized into a double helical arrangement, a “double spiral staircase.” Basic residues interacting with the phosphodiester DNA backbone are highlighted. Subunits D and E, the β-subdomains of which do not interact with DNA, have been removed for clarity. (*B*) Schematic representation of *A*. (*C*) Top view of *A* with all six subunits. Subunits D and E are far away from the DNA. (*D*) All direct interactions between DNA and basic residues in loops (staircases) I and II. Each of the four interacting subunits (indicated by colors and letters) contacts two phosphates, implying a translocation of exactly 2 bp per step.

The DNA density is very well defined for 20 bp, traversing the pore from one end to the other. Density for the nucleobases is the result of averaging over all four possibilities because FtsK’s DNA translocase activity is not sequence specific, and hence no sequence was assigned to the DNA. Additional map density extended on both sides but had lower quality presumably due to DNA flexibility outside the pore. Contrary to the nucleotide-free, ADP, and AMPPNP states, in the translocating state DNA makes extensive contacts with four subunits in the β-ring, F, A, B, and C, adopting conformations 6, 1, 2, and 3 (Fig. 3). The most prominent DNA contacts in all four subunits involve basic residues protruding into the pore from two loops contacting the phosphodiester backbone on both strands of the minor groove. Loop I binds to two phosphates from one strand, exclusively through contacts facilitated by K657 and R661. Similarly, two phosphates of the other DNA strand are recognized by loop II through R632 and to a lesser extent K643. H-bonds with additional loop II residues S634, V635, and G640 reinforce this interface. The conformational heterogeneity around the ring positions these loops in two helical paths, or “spiral staircases,” following the DNA’s helix precisely (Movie S1). This arrangement is reminiscent of the single spiral staircases observed in ring-shaped helicases (24). In FtsK, conformation 3 (subunit C) produces a DNA interface at the bottom of the staircase, followed by the two other ATPγS-bound conformations 2 and 1 (subunits B and A). The path is terminated by the ADP-bound conformation 6 (subunit F) at the top. Conformations 5 and 4 (subunits E and D) are not bound to DNA, allowing two subunit positions for the gradual transition from the top of the spiral staircase to the bottom, resetting the subunit conformation. The gradual decrease in α- to β-angle from conformation 3 to 1 creates a smooth transition, which is broken somewhat by conformation 6 and its larger step size. Curiously, this irregularity distorts the double helix by widening the minor groove by up to 25% (from 11.8 to 14.7 Å) from canonical B-form DNA (*SI Appendix*, Fig. S9*A*).

### DNA Exit from the Hexameric Pore.

Several contacts are made with the DNA at the pore’s exit, at the position of the α-ring (*SI Appendix*, Fig. S9*B*). These interactions are mediated by interactions with K377 and R380. However, because of the largely preserved rotational symmetry of the α-ring, these residues form a flat ring, almost perpendicular to the DNA’s long axis. This configuration prevents tracking of the DNA helicity, but might allow subunits on both sides of the DNA to maintain their alignment with the pore’s axis as it is exiting. Indeed, one DNA strand is contacted by the α-subdomains of protomers in conformations 1 and 6 (subunits A and F), while the other is by conformation 4 (subunit D), the β-subdomain of which is disengaged from DNA.

### Translocation Mechanism.

**Fig. 4.**
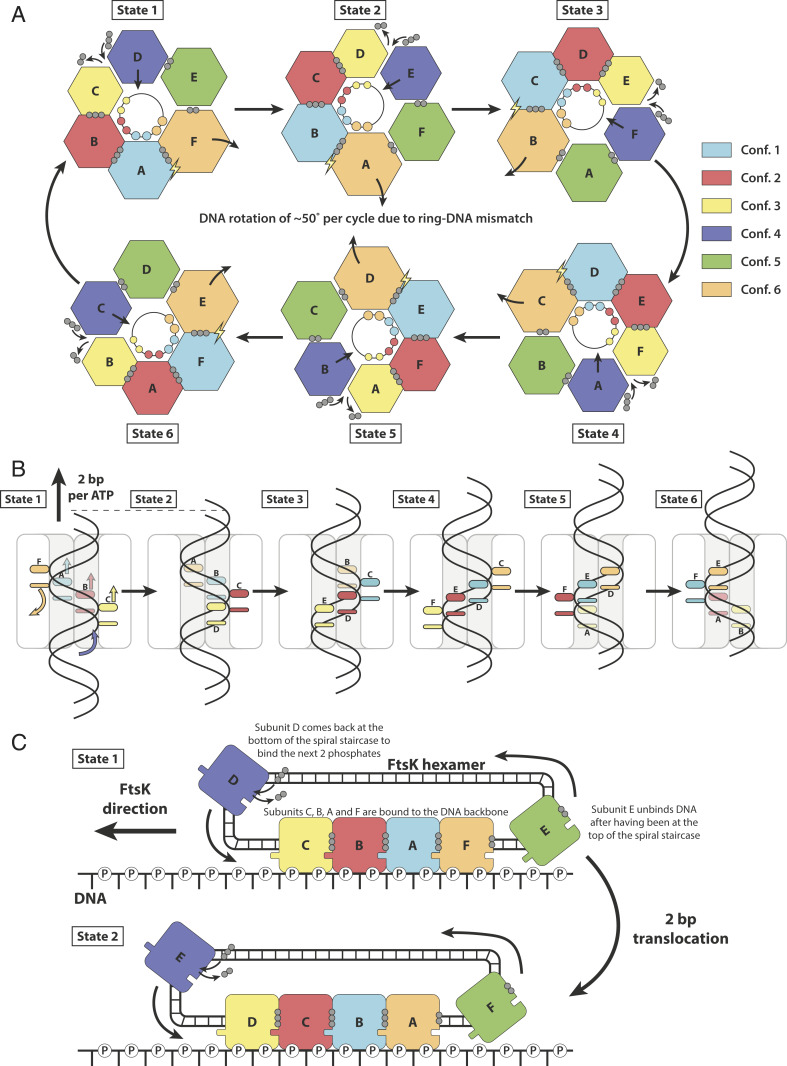
Model for double-stranded DNA translocation. (*A*) The six conformations rotate around the ring. As each subunit advances one-sixth further along the ATPase reaction cycle, it adopts the conformation of the subunit next to it. The conformations, but not the subunits, rotate clockwise around the ring (viewed from the α-ring). Because the DNA is helical, rotational adaption of the six conformations leads to 2-bp translocations at each step. Since DNA has 10.5 bp per turn, there needs to be a rotation of FtsK against the DNA of 51.4 ° per cycle of six hydrolyzed ATP and 12 bp translocated. Spheres: phosphates; lightning bolt: hydrolysis-competent state. (*B*) DNA translocation through the FtsK_αβ_ pore. In each state, four subunits bind dsDNA through two loops, organized into two spiral staircases. (*C*) The translocation mechanism understood as circularized filament treadmilling. As in cytomotive filaments, the spiral staircase along the DNA backbone is extended at one end (conformation 3, yellow, binding DNA last), and shortened at the other end (conformation 6, orange, detaching next). Non–DNA-binding conformations 4 and 5 circularize the filament into a ring since they close the conformational wave, resetting conformation 6 into 3.

Because the subunits seen in the asymmetric ATPγS FtsK_αβ_ structure appear to describe an entire ATP hydrolysis and conformational cycle, the structure directly suggests a mechanism for dsDNA translocation by FtsK_αβ_ that involves rotating the conformational states around the ring. Hence our mechanistic model has at its center a concerted, simultaneous conformational change of all subunits, at least in its most idealized form. At each step, all subunits change conformation and advance along the nucleotide hydrolysis cycle by one-sixth of the entire cycle and more-or-less simultaneously (Fig. 4*A* and Movies S2 and S3). The nucleotide states around the ring indicate that the reaction cycle advances around the ring clockwise when looking from the α-ring side (Fig. 4*A*), as postulated previously (13). Conformation 1 (subunit A in the structure) is therefore competent for ATP hydrolysis and the next to convert ATP into ADP. Importantly, the right-handedness of the DNA and the direction of hydrolysis around the ring suggests a translocation direction of the DNA from the β-ring toward the α-ring (Fig. 4*B*), and this is in agreement with previous data (10, 12, 13).

In each step, all subunits change conformation in a concerted manner, acquiring the next conformation in the cycle. As a result, the subunit at the top of the spiral staircase (subunit F) disengages from dsDNA, switching from conformation 6 to 5. At the bottom of the staircase, another subunit contacts the dsDNA phosphodiester backbone and binds ATP, shifting from conformations 4 to 3 (subunit D in the structure). Every catalytic cycle adds one ATP-bound subunit at the bottom end of the spiral staircase, while removing one ADP-bound at the top, similar to the treadmilling of cytomotive protein filaments, but in a closed circular arrangement (Fig. 4*C* and Movie S3). Using the treadmilling analogy, conformations 4 and 5, resetting the β-subdomains from the top to the bottom for renewed binding, represent the recycling of subunits from one end to the other.

The arginine finger, as in other ring-shaped ATPases of similar types (25), ensures coordination between the different states. The concerted conformational changes within the spiral staircase all participate in DNA translocation, with each of the four subunits remaining associated with the same two phosphates during the process (Fig. 4*B*). In our model, 2 bp of the DNA are translocated per catalytic step and 12 bp by a full cycle around the ring (Fig. 4*B*), as postulated before (13, 23, 26). This must be accompanied by a 51.4 ° anticlockwise rotation of dsDNA per cycle and 12 bp (∼4 °/bp) and, hence, supercoiling (or protein rotation) to compensate for the mismatch with the roughly 10.5 bp per canonical DNA turn.

The α-ring, although probably not actively involved in translocation, may be essential for the high processivity observed by FtsK. We propose that the α-subdomain's almost symmetrical arrangement throughout the ring and reaction cycle ensures that the ring does not open during translocation. In addition, the α-ring seems to passively guide the positioning of dsDNA through the pore for optimal interactions with the spiral staircases, acting like an aperture.

Our model for dsDNA translocation by FtsK_αβ_ recapitulates important features of the previous mechanistic models for ring-shaped helicases that act as single-stranded DNA motors and in particular features from superfamily 5, such as Rho helicase (17, 24). However, the double-stranded nature of the much more rigid dsDNA substrate of FtsK requires larger movements of the ATPase subdomains and a second spiral staircase which enables 3′ to 5′ translocation of the second strand, to our knowledge a unique feature among RecA-type DNA motors. In addition, the great speed of FtsK seems to require an additional processivity device, the nondeformable α-ring. Further studies on other members of the FtsK/HerA family of proteins (27) will be essential to extend our insights to dsDNA translocases involved in sporulation, conjugation, dsDNA break repair, and viral DNA packaging and to determine which features make FtsK such an outstandingly fast double-stranded DNA motor.

## Materials and Methods

### Cloning and Expression.

The gene coding for FtsK from *P. aeruginosa* strain ATCC 15692 (residues 247 to 728, UniProt entry Q9I0M3) was amplified and cloned into vector pHis17 ahead of a C-terminal KLHHHHHH tag by Gibson assembly (13). *Escherichia coli* C41(DE3) cells were transformed by electroporation with the resulting plasmid and plated on a Tryptone Yeast Extract (TYE) agar plate supplemented with 100 µg⋅mL^−1^ ampicillin. Then, a 50-mL 2xTY preculture was grown overnight at 37 °C from a single colony in presence of 100 µg⋅mL^−1^ ampicillin. After centrifugation (10 min, 3,220 × *g* at 4 °C), the cells were used to inoculate 6 L of 2xTY medium supplemented with 100 µg⋅mL^−1^ ampicillin. The culture was grown at 37 °C until an OD_600_ of ∼0.6 was reached. Protein expression was induced with 1 mM isopropyl β-d-1-thiogalactopyranoside (Anatrace) at 25 °C for 6 h. The cells were harvested by centrifugation (20 min, 5,300 × *g* at 4 °C) and stored at −20 °C.

### Purification.

The cell pellet was resuspended in 150 mL buffer (50 mM Tris, 150 mM NaCl, pH 7.0), complemented with DNase I (Sigma), RNase A (Sigma), and ethylenediaminetetraacetic acid (EDTA)-free protease inhibitor tablets (Roche). Cells were lysed with a cell disruptor (Constant Systems) at 25 kilo pounds per square inch, and the lysate was centrifuged for 45 min at 142,000 × *g* in a Type 45 TI rotor (Beckman) at 4 °C. Imidazole (50 mM) was added to the supernatant before loading onto a 5-mL HisTrap HP column (GE Healthcare). The column was washed with buffer A1 (50 mM Tris, 150 mM NaCl, 50 mM imidazole, pH 7.0), and the protein was eluted with step-wise increments of buffer A2 (50 mM Tris, 150 mM NaCl, 1 M imidazole, pH 7.0). Fractions containing the protein were pooled and loaded onto a 5-mL HiTrap Heparin HP column (GE Healthcare). The column was washed with buffer B1 (50 mM Tris, 150 mM NaCl, pH 7.0) before eluting the protein with step-wise increments of buffer B2 (50 mM Tris, 1 M NaCl, pH 7.0). Fractions were pooled and concentrated to 2 mL in a Vivaspin 20 concentrator (polyethersulfone [PES] membrane), 50-kDa molecular weight cutoff, Sartorius). The concentrate was loaded onto a Sephacryl 16/60 S-300 HR column (GE Healthcare) and eluted in buffer C (25 mM Tris, pH 7.5). The protein was concentrated with a Vivaspin 2 concentrator (PES membrane, 50-kDa molecular weight cutoff, Sartorius) before being flash-frozen in liquid nitrogen for storage at −80 °C.

### NMR Spectroscopy.

The 1D ^31^P spectra were recorded at 202.4 MHz, 298 K, on an Avance II-500 spectrometer (Bruker) equipped with a broadband cryoprobe. Data acquisition time was 3.3 min in each spectrum, with a 2.9-s interscan delay and 0.15 Hz/point digital resolution in the processed data. For each reaction, samples of 1.5 µM FtsK_αβ_, 180 nM DNA, in 25 mM Tris, 5% (vol/vol) D_2_O, pH 7.5, were prepared. Reactions were started with the addition of 1 mM nucleotide and 2 mM MgCl_2_ (or 2 mM nucleotide and 4 mM MgCl_2_ for time series). Under these conditions, ATP was fully hydrolyzed by FtsK_αβ_ during the 7-min dead time between sample insertion and the end of data collection of the first spectrum. Normalized peak integrals for the α-phosphorus measured for the time series spectra were used to estimate the percentage of each nucleotide in the sample over time.

### Cryo-Electron Microscopy.

Mixtures of 0.7 mg⋅mL^−1^ (13 µM) FtsK_αβ_, 1.5 µM 45-bp dsDNA (sequence: CGC​AGG​AAA​AAT​AGC​GAT​TTG​AAG​GAT​TCG​ACC​ACC​GCG​AGC​CAT) in 25 mM Tris, pH 7.5, were prepared on ice. After 2 min incubation with final concentrations of 2 mM ATPγS and 4 mM MgCl_2_, 3 µL of the samples was added to glow-discharged R1.2/1.3 AU 300 mesh Quantifoil grids. The grids were blotted for 2 s at force −5 and flash-frozen into liquid ethane after 5 s waiting time, using a Thermo Fisher Vitrobot Mark IV. Nucleotide-free, ADP, and AMPPNP samples were prepared similarly with incubation times up to 15 min. The FtsK_αβ_-dsDNA complex in the presence of ATPγS was imaged on a Titan Krios electron microscope from the Electron Bio‐Imaging Centre (eBIC) at Diamond Light Source. A total of 3,300 micrographs were collected using EPU software on a K2 camera in counting mode at a nominal magnification of 130,000 x, with a resulting pixel size of 1.048 Å. Each movie was recorded for 12 s, with a total dose of ∼43 e^−^/Å^2^ over 48 fractions, within a nominal defocus range of −3.5 to −2.0 µm. The other FtsK_αβ_-dsDNA complex samples (nucleotide-free, ADP and AMP-PNP) were imaged similarly on an in-house Titan Krios microscope. Micrographs were collected on a K2 camera in counting mode, at pixel sizes ranging from 1.08 to 1.145 Å, and total doses from ∼39 to ∼49 e^−^/Å^2^.

### Cryo-EM Data Processing.

All data were processed in Relion 3.0 (28). For the FtsK_αβ_-dsDNA-ATPγS sample, dose-weighted micrographs were motion corrected with MotionCor2 (29), and their contrast transfer function (CTF) parameters were estimated with CTFFIND 4.1 (30). Using an estimated resolution cutoff of 6 Å, 49 images were discarded from the next steps. A total of 414 particles were manually picked and 2D classified. Using these 2D classes as references, a total of 1,510,109 particles were picked automatically and extracted. After 2D classification, 1,094,683 particles were selected and sorted into four classes through 3D classification using as a reference a 40-Å low-passed filtered map of the symmetrical FtsK_αβ_-ring from crystallography (Protein Data Bank [PDB] ID 2IUU, no DNA) and a 180-Å circular mask. While one class was discarded for its poor quality, the three others were separately reconstructed (with a mask including just the FtsK ring and the DNA), and each class was used for a round of further 3D classification without alignment. The resulting classes were again reconstructed, and those with the highest resolutions and best DNA density were further improved using per particle CTF correction, Bayesian polishing, and postprocessing. This resulted in three maps, one in a highly asymmetric state (later assigned to a translocating state) at 3.6-Å resolution (map III.E) (*SI Appendix*, Fig. S3), and two almost symmetrical states (later assigned to a stalled state), one of which with dsDNA visible all along the pore (4.34-Å resolution, III.B), while in the other one dsDNA is found only in the upper half of the pore, toward the pore’s exit (3.99-Å resolution, II.A). During processing, many other classes appeared that represented only small variations of each of these three, with often less-defined DNA or lower resolution for some subunits. These were not used for final refinements as their inclusion decreased quality of the generated maps.

FtsK_αβ_-dsDNA samples in the nucleotide-free, ADP, or AMPPNP states were processed similarly in the early stages with 393,540; 240,870; and 265,603 particles selected, respectively, after 2D classification. However, after 3D classification, EM maps with a well-resolved symmetrical ring but a featureless dsDNA were obtained for each dataset. Given the small size of the DNA compared to the protein, and the absence of any visible conformational variation among the subunits forming the ring, it is likely that the particles’ orientations could not be determined accurately enough around the pseudo-sixfold axis of the protein, and hence the DNA, which has no symmetry, was poorly defined. Further processing steps involved FtsK_αβ_ signal subtraction and 3D classification on the DNA itself (using a 30-Å low-passed filtered map generated from a dsDNA model). Classes with visible DNA grooves were selected (56,904; 29,563; and 28,958 particles for FtsK_αβ_-dsDNA, respectively, without any nucleotide, with ADP, or with AMP-PNP), and were refined and postprocessed based on the full particles signal, to reconstruct the complexes with only partial recovery of the dsDNA’s detailed features.

### Model Building and Refinement.

The crystal structure of FtsK_αβ_ (PDB ID 2IUU) was docked into map III.E using UCSF Chimera (31) to serve as a starting model for atomic model building of the translocating complex. For the dsDNA, a 20-bp B-form dsDNA model was generated in Coot (32) and fitted manually inside the map. Subsequently, the model underwent several rounds of manual rebuilding in Coot and real-space refinement in Phenix (33). The model’s stereochemistry and map fit were validated with Molprobity and EMRinger (34, 35), respectively. Similar procedures were performed for FtsK_αβ_-dsDNA–stalled states associated with maps II.A and III.B.

### Model and Morph of dsDNA Translocating through FtsK_αβ_.

The six conformations around the FtsK_αβ_ ring each represent a snapshot of one-sixth of the complete hydrolysis cycle of each subunit. They hence represent a wave-like continuous conformational change around the ring. By changing each subunit’s conformation into the conformation of the next conformation in the reaction cycle (the subunit next to it in the ring), simultaneously for all subunits, an atomic morph of a complete translocation cycle can be generated through interpolation between each snapshot (six ATP hydrolyzed, 12 bp translocated). Briefly, each protein-DNA state was generated by incremental 60 ° (clockwise from top) rotation of the entire FtsK_αβ_-dsDNA complex. Subunits were renamed afterward so that every protomer keeps the same chain ID for a given position in the ring. DNA translocation was performed by extending the dsDNA by 2 bp at one end (exit side), shortening it by the same length on the other side at each step and renumbering the bases so that each strand’s 5′ end always starts with base number 1. Intermediate states for Movies S2 and S3, between the six key states generated in this way, were derived by morphing in PyMOL. Together, these steps allowed visualizing the slight rotation of dsDNA as well as its continuous slight deformation as it traverses the channel (see top views in Movies S2 and S3).

### 

## Supplementary Material

Supplementary File

Supplementary File

Supplementary File

Supplementary File
