# Supplementary material for: FtsK in motion reveals its mechanism for double-stranded DNA translocation
Source: Proc Natl Acad Sci U S A. 2020 Jun 8;117(25):14202–8. doi: 10.1073/pnas.2001324117 (PMC7321959; doi:10.1073/pnas.2001324117)
Supplement: Supplementary File [file pnas.2001324117.sapp.pdf]

PNAS
www.pnas.org

Supplementary Information for

FtsK in motion reveals its mechanism for double-stranded DNA translocation

Nicolas L. Jean[a], Trevor J. Rutherford[a], Jan Löwe[a1]

[a] MRC Laboratory of Molecular Biology, Cambridge, UK.

[1] To whom correspondence should be addressed. jyl@mrc-lmb.cam.ac.uk

This PDF file includes:

Figures S1 to S9
Tables S1 to S2
Legends for Movies S1 to S3

Other supplementary materials for this manuscript include the following:

Movies S1 to S3

www.pnas.org/cgi/doi/10.1073/pnas.2001324117

Supplementary Figures

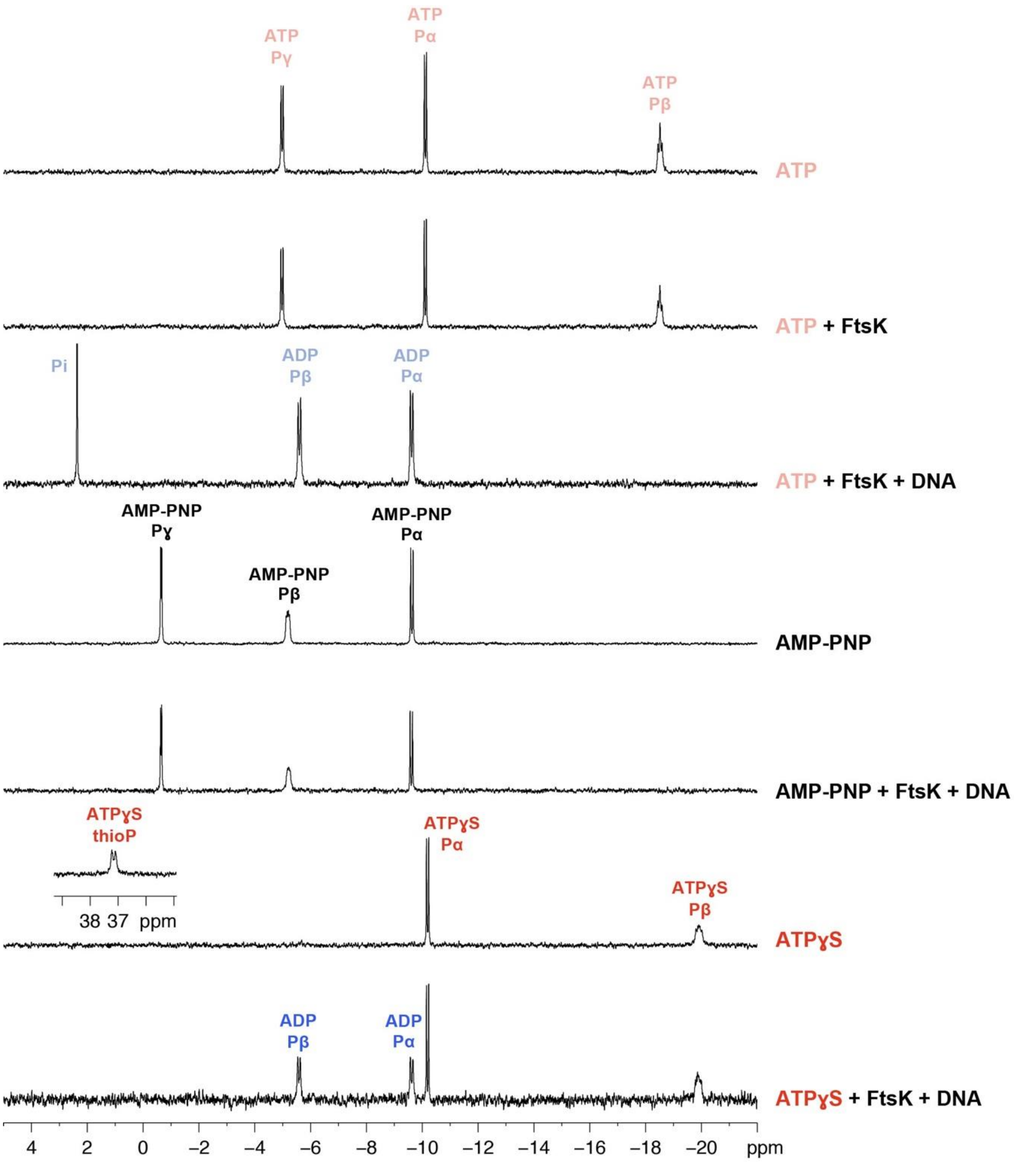

Figure S1. Nucleotide hydrolysis activity of $FtsK_{\alpha\beta}$ measured by NMR. 1D ^{31}P NMR spectra of different mixtures of $FtsK_{\alpha\beta}$, dsDNA and nucleotides. Samples were prepared as detailed in Materials & Methods. Spectra were collected seven minutes after nucleotide addition. ATP (fast) and ATPγS (slow) were hydrolysed by $FtsK_{\alpha\beta}$ only in the presence of DNA, but not AMPPNP.

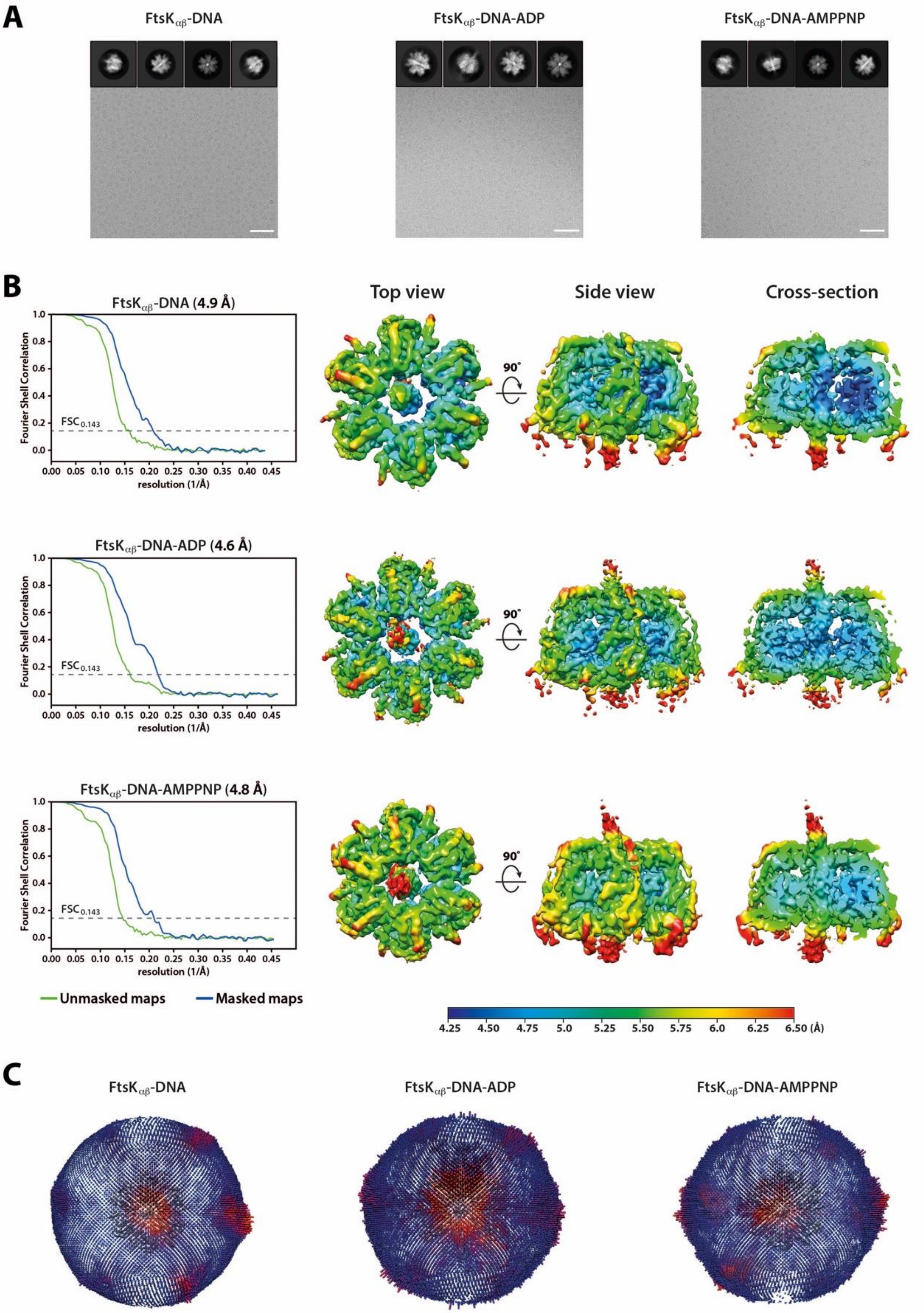

A
FtsKαβ-DNA
FtsKαβ-DNA-ADP
FtsKαβ-DNA-AMPPNP
B
FtsKαβ-DNA (4.9 Å)
FtsKαβ-DNA-ADP (4.6 Å)
FtsKαβ-DNA-AMPPNP (4.8 Å)
Top view
Side view
Cross-section
90°
Fourier Shell Correlation
resolution (1/Å)
FSC0.143
1.0
0.8
0.6
0.4
0.2
0.0
0.00 0.05 0.10 0.15 0.20 0.25 0.30 0.35 0.40 0.45
Unmasked maps
Masked maps
4.25 4.50 4.75 5.0 5.25 5.50 5.75 6.0 6.25 6.50 (Å)
C
FtsKαβ-DNA
FtsKαβ-DNA-ADP
FtsKαβ-DNA-AMPPNP

Figure S2. Validation of the $FtsK_{\alpha\beta}$-DNA maps in the nucleotide-free, ADP- and AMPPNP- states. (*A*) Representative cryo-EM micrographs from each of the 3 datasets with typical 2D classes (top). (*B*) Resolution estimation of cryo-EM maps for $FtsK_{\alpha\beta}$-DNA complexes. Left panels show the Fourier shell correlation (FSC) curves for masked (blue) and unmasked (green) maps using gold standard half-maps. The $FSC_{0.143}$ threshold for resolution estimation is indicated by a dotted grey line. The panels on the right show top, side and cross-sectioned views of the complexes, coloured by local resolution as determined by Relion 3.0. (*C*) Distribution of Euler angles for each of the 3 maps as generated by Relion 3.0.

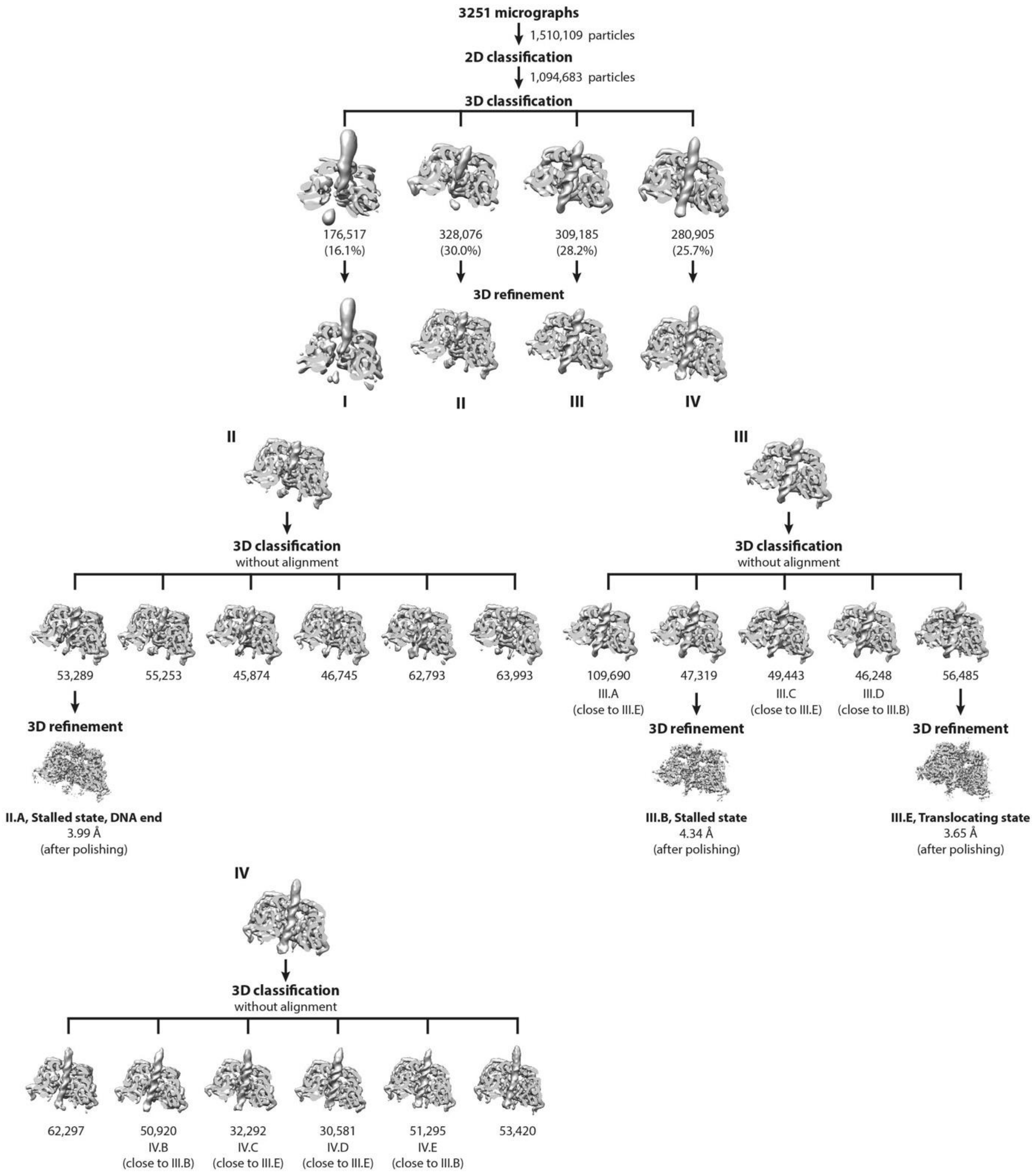

Figure S3. Data processing workflow of the FtsK$_{\alpha\beta}$-dsDNA + ATPγS dataset. Processing was performed in Relion 3.0. After 3D classification, good particles were sorted into three main classes (II, III and IV). Further 3D classification identified three classes (II.A, III.B and III.E) that were better resolved and had very well-defined DNA density. The number of particles in each class is indicated underneath.

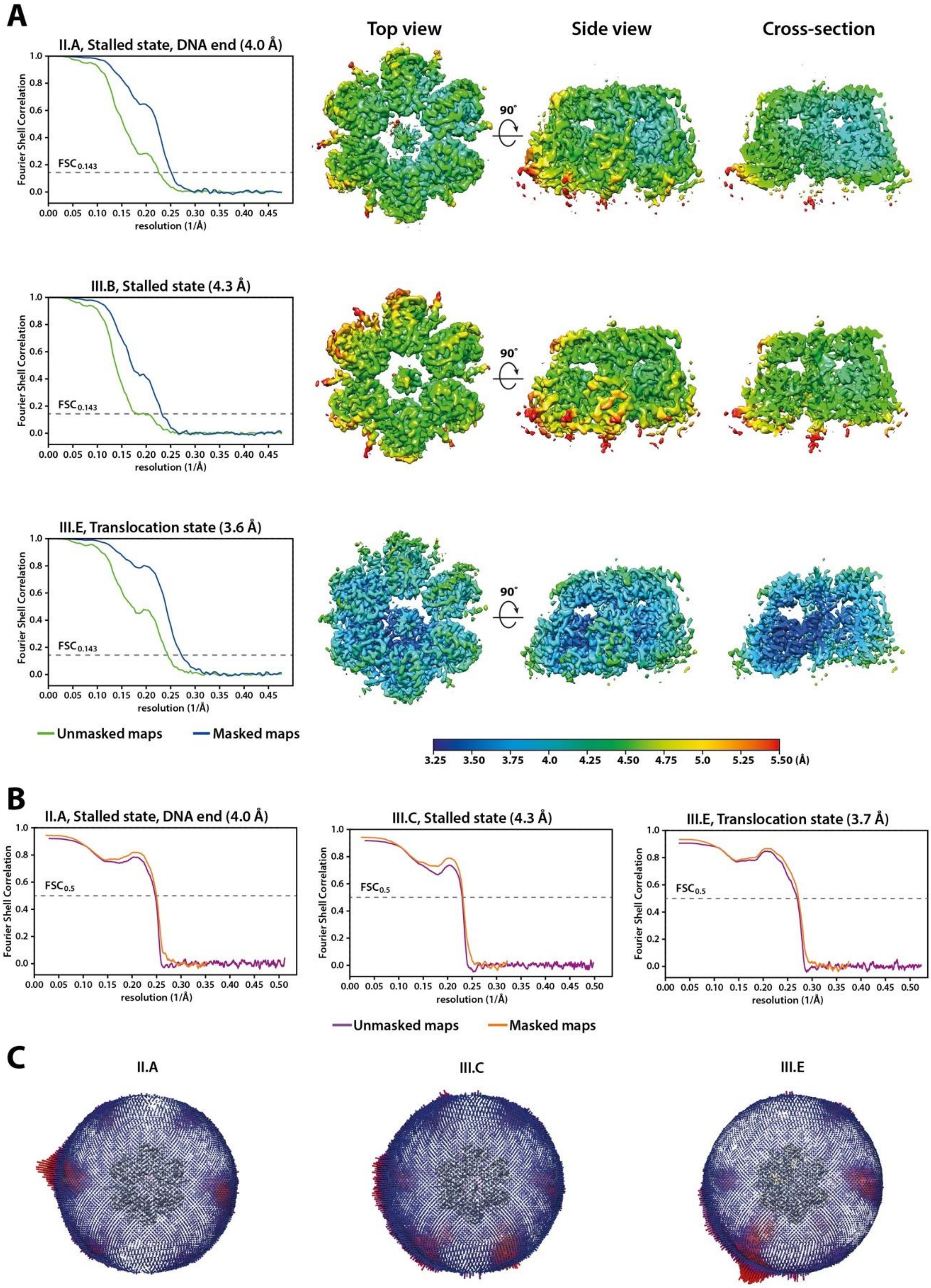
A
II.A, Stalled state, DNA end (4.0 Å)
III.B, Stalled state (4.3 Å)
III.E, Translocation state (3.6 Å)
Fourier Shell Correlation
resolution (1/Å)
FSC0.143
Top view
Side view
Cross-section
90˚
Unmasked maps
Masked maps
3.25 3.50 3.75 4.0 4.25 4.50 4.75 5.0 5.25 5.50 (Å)
B
II.A, Stalled state, DNA end (4.0 Å)
III.C, Stalled state (4.3 Å)
III.E, Translocation state (3.7 Å)
FSC0.5
Unmasked maps
Masked maps
C
II.A
III.C
III.E

Figure S4. Validation of structural data for the FtsK$_{\alpha\beta}$-dsDNA complex in the ATPγS sample. (*A*) Resolution estimation of FtsK$_{\alpha\beta}$-dsDNA complexes. Left panels show the Fourier shell correlation (FSC) curves for masked (blue) and unmasked (green) maps using gold standard half-maps. The $FSC_{0.143}$ threshold for resolution estimation is indicated by a dotted grey line. Panels on the right represent top, side and cross-sectioned views of the complexes, coloured by local resolution as determined by Relion 3.0. (*B*) Map-to-model FSCs. Curves in orange are for masked maps and purple curves represent unmasked maps. The $FSC_{0.5}$ threshold is represented by a dotted grey line. Resolutions estimated from each FSC curves are indicated above and closely match those estimated for the maps the models are derived from and have been refined against. (*C*) Distribution of Euler angles as generated by Relion 3.0.

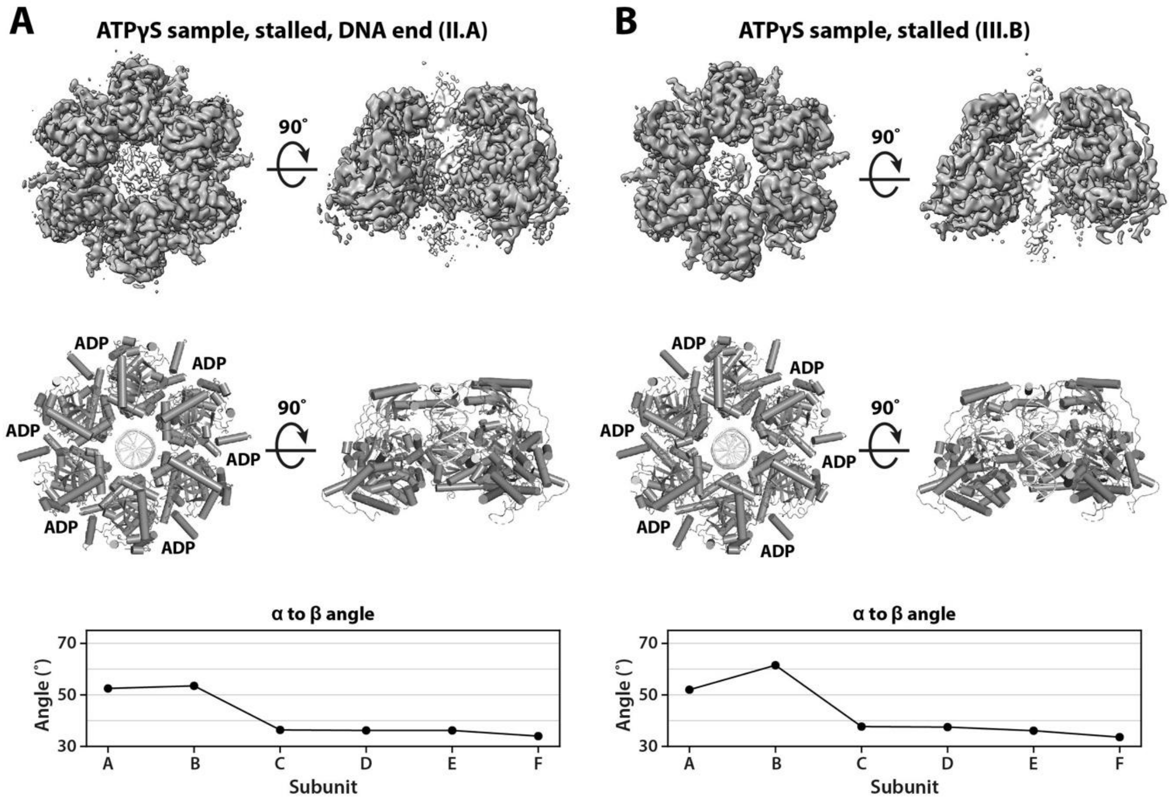

Figure S5. Further structures of FtsK$_{\alpha\beta}$-dsDNA complexes from the ATPγS sample. (*A*) and (*B*) show maps and atomic models of FtsK$_{\alpha\beta}$ with partial (DNA end) or full DNA signals, respectively. The graphs in lower panels illustrate the angles measured between the α and β subdomains across the 6 subunits. Because the structures do not contain nucleotide states of a full hydrolysis cycle around the ring, the angle distribution does not show a wave function (as in Figure 2*A*, bottom; and hence the ring is much less asymmetric) and the DNA density is truncated and/or less well defined, we propose that these structures represent stalled states of the motor.

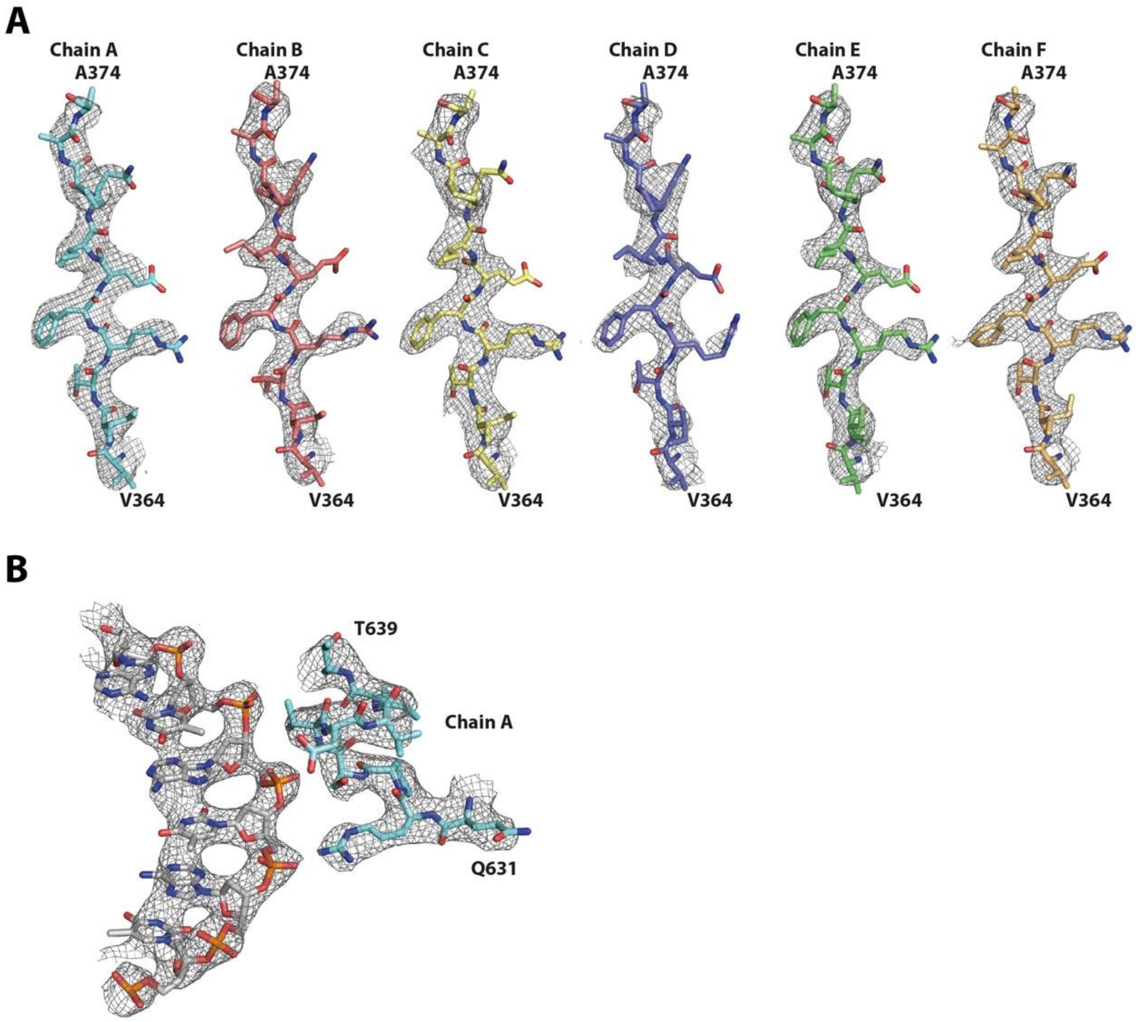

Figure S6. Representative cryo-EM map details of the FtsK$_{\alpha\beta}$-dsDNA complex in the translocating state. (*A*) Residues V364 to A374 of all six subunits and (*B*) the interaction between loop I from chain A and one DNA strand. The refined atomic models for these regions are shown superimposed.

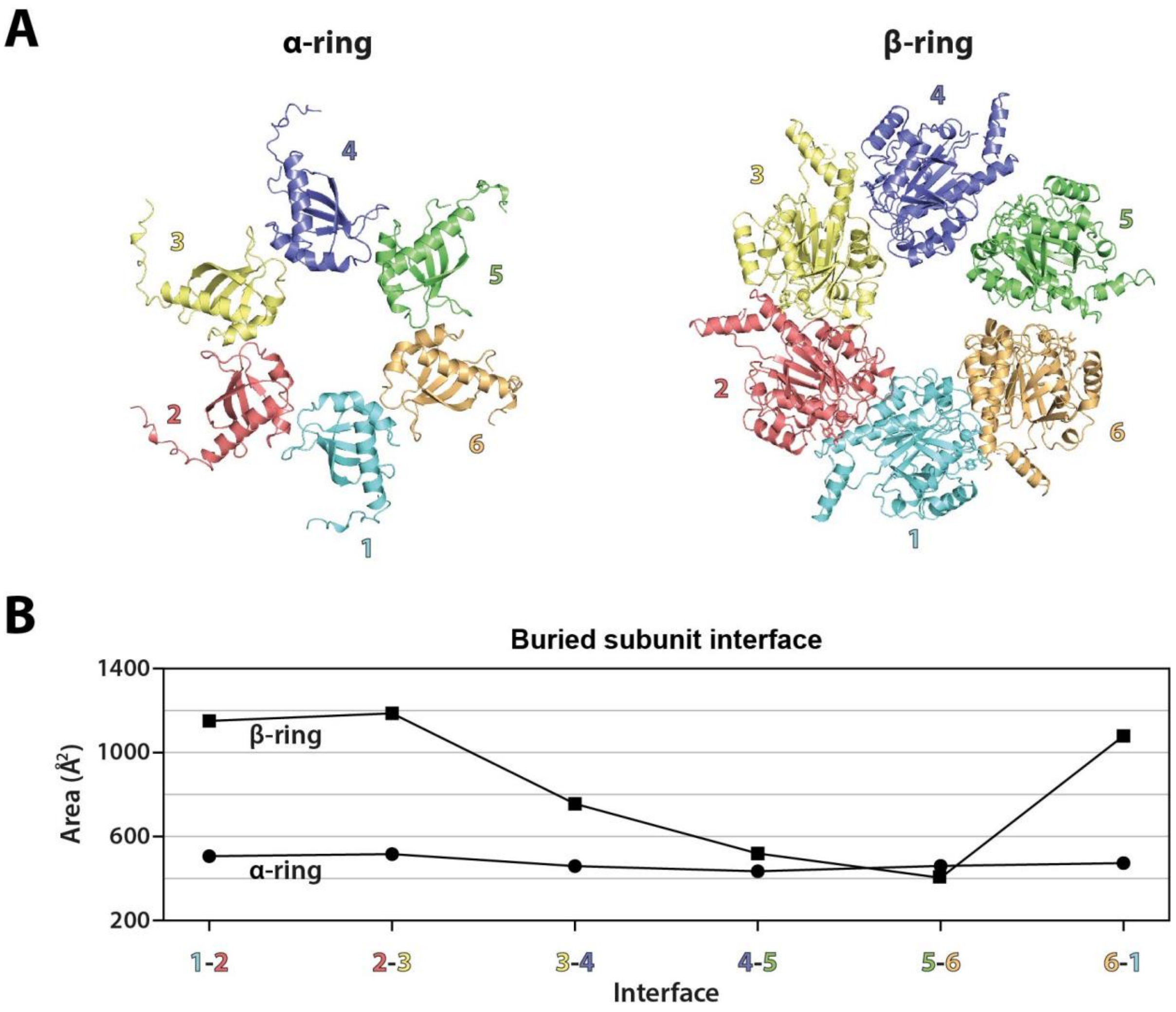

Figure S7. Molecular packing analysis of the translocating FtsK$_{\alpha\beta}$-dsDNA complex in the ATPγS sample. (*A*) Structures of the isolated α (left) and β (right) rings. Subunit conformations are indicated by numbers shown next to each structure. (*B*) Buried subunit surfaces in the interfaces along each ring. Note that the α ring is more-or-less symmetric, whereas the β ring is not.

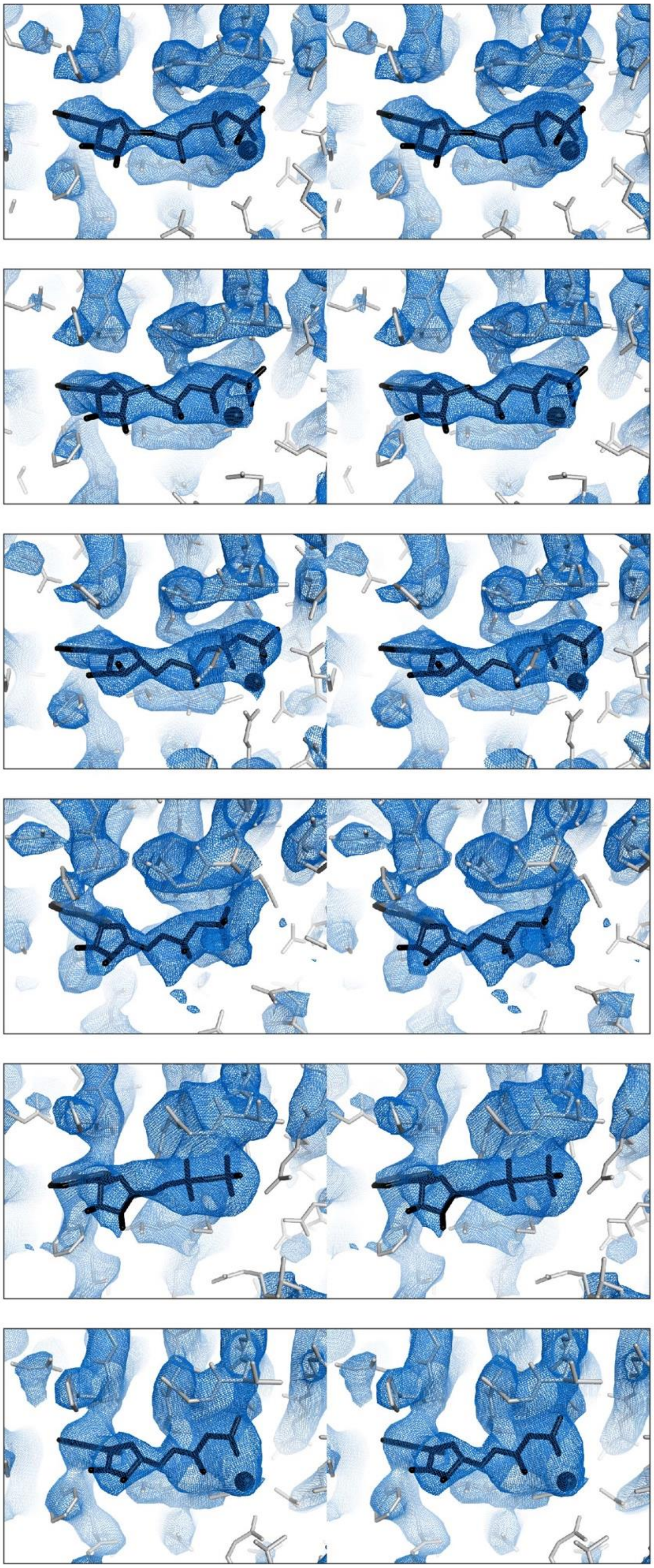

A-F ATPγS

B-A ATPγS

C-B ATPγS

D-C Nucleotide exchange

E-D ADP

F-E ADP

Figure S8. Stereo views of cryo-EM densities around each nucleotide from the $FtsK_{\alpha\beta}$-dsDNA complex (translocating state). The atomic model of the assigned nucleotides and neighbouring residues are superimposed to the cryo-EM maps in black and light grey respectively.

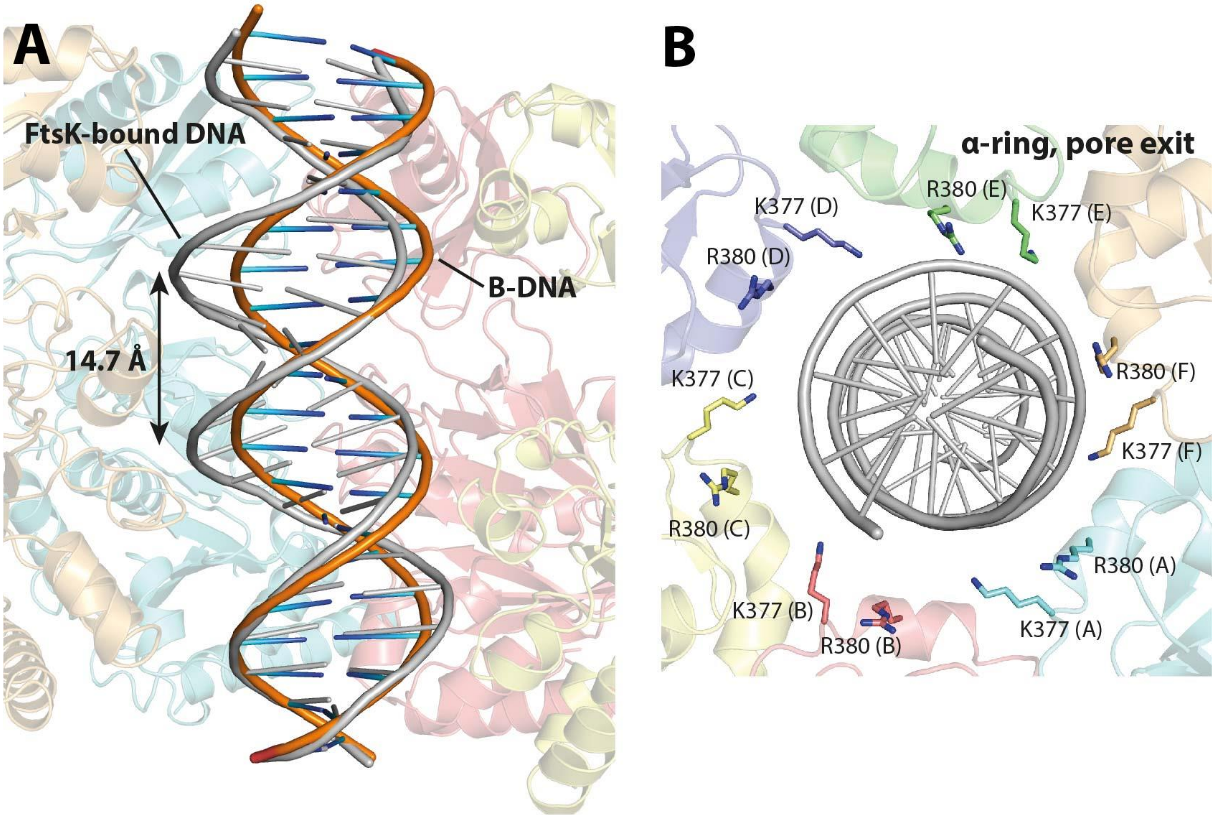

Figure S9. Atomic details of $FtsK_{\alpha\beta}$-dsDNA interactions in the translocating state. (*A*) DNA distortion by $FtsK_{\alpha\beta}$. Interaction with $FtsK_{\alpha\beta}$ widens the DNA's minor groove by up to 2.9 Å (+25%) (grey) compared to canonical B-form DNA (orange). (*B*) Top view of DNA pore formed by the α-ring. Residues K377 and R380 on each subunit create a positively charged ring at the pore's exit, shown here as sticks.

Supplementary Tables

Table S1. Cryo-EM data collection and processing of nucleotide-free, ADP- and AMPPNP-bound $FtsK_{\alpha\beta}$-dsDNA complexes.

	**$FtsK_{\alpha\beta}$-DNA (EMD 10403)**	**$FtsK_{\alpha\beta}$-DNA-ADP (EMD 10404)**	**$FtsK_{\alpha\beta}$-DNA-AMPPNP (EMD 10405)**
**Data collection**			
**Microscope**	Titan Krios	Titan Krios	Titan Krios
**Voltage (kV)**	300	300	300
**Detector**	K2 Summit	K2 Summit	K2 Summit
**Nominal magnification**	105,000	130,000	105,000
**Pixel size (Å)**	1.145	1.08	1.10
**Total electron fluence (e-/Å^2)**	43.8	39.4	48.6
**Defocus range (μm)**	-3.5 to -2	-3.5 to -2	-3.5 to -2
**Data processing**			
**Micrographs**	742	771	830
**Extracted particles**	397,748	514755	311,615
**Refined particles**	393,540	240,870	265,603
**Final particles**	56,904	29,563	28,958
**Map resolution (Å)**	4.91	4.63	4.80
**FSC threshold**	0.143	0.143	0.143
**Map resolution range (Å)**	4.35 to 7.0	4.55 to 7.0	4.6 to 7.1

Table S2. Cryo-EM data collection, processing and refinement statistics of structures from $FtsK_{\alpha\beta}$-dsDNA plus ATPγS.

	**$PaFtsK_{\alpha\beta}$-DNA-ATPγS Translocation state (EMD 10399, PDB 6T8B)**	**$PaFtsK_{\alpha\beta}$-DNA-ATPγS Stalled state (EMD 10400, PDB 6T8G)**	**$PaFtsK_{\alpha\beta}$-DNA-ATPγS Stalled state, DNA end (EMD 10402, PDB 6T8O)**
**Data collection**			
**Microscope**	Titan Krios	Titan Krios	Titan Krios
**Voltage (kV)**	300	300	300
**Detector**	K2 Summit	K2 Summit	K2 Summit
**Nominal magnification**	130,000	130,000	130,000
**Pixel size (Å)**	1.048	1.048	1.048
**Total electron fluence (e-/Å^2)**	42.95	42.95	42.95
**Defocus range (µm)**	-3.5 to -2	-3.5 to -2	-3.5 to -2
**Data processing**			
**Micrographs**	3,300	3,300	3,300
**Extracted particles**	1,510,109	1,510,109	1,510,109
**Refined particles**	1,094,683	1,094,683	1,094,683
**Final particles**	56,485	47,319	53,289
**Map resolution (Å)**	3.65	4.34	3.99
**FSC threshold**	0.143	0.143	0.143
**Map resolution range (Å)**	3.4 to 5.0	4.1 to 5.8	3.8 to 5.4
**Refinement**			
**Initial model used (PDB code)**	2IUU	2IUU	2IUU
**Map sharpening B-factor (Å^2)**	-90.9	-147.3	-120.1
**Model composition**			
**Non-hydrogen atoms**	19,077	18,875	18,806
**Residues**	2370 (FtsK); 40 (DNA)	2367 (FtsK); 32 (DNA)	2374 (FtsK); 26 (DNA)
**Ligands**	10	6	6
**Map correlation coefficient**	0.82	0.83	0.82
**R.m.s deviations**			
**Bond lengths (Å)**	0.004	0.008	0.006
**Bond angles (˚)**	0.720	1.138	1.242
**Validation**			
**MolProbity score**	2.11	1.95	1.61
**Clashscore**	13.97	7.19	4.39
**Poor rotamers (%)**	0.0	0.15	0.20
**Ramachandran plot**			
**Favored (%)**	92.80	89.80	94.21
**Allowed (%)**	7.20	10.20	5.79
**Disallowed (%)**	0.00	0.00	0.00
**EMRinger score**	2.47	1.45	1.99

Legends for Supplementary Movies

Movie S1. Recognition of dsDNA by a double 'spiral staircase' of DNA-interacting loops is facilitated by $FtsK_{\alpha\beta}$'s conformational diversity around the ring. Each subunit is coloured differently according to their conformation. The translocating III.E structure contains three distinct nucleotide states (ATPγS, ADP, and nucleotide exchange). Basic residues from loops I and II are interacting with the minor groove of the double-stranded DNA (interacting residues coloured in red) and are arranged as two spiral staircases containing four of the six subunits. Disengaged residues (K643 from conformation 1, residues from subunits D and E) are depicted in grey.

Movie S2. Model for dsDNA translocation by $FtsK_{\alpha\beta}$. The movie shows the concerted conformational changes during the catalytic cycle to translocate dsDNA. Each subunit goes through all six stages of hydrolysis and their associated conformational states, meaning that the conformations rotate around the ring (depicted by colours). The DNA rotates only slowly against the subunits because of a symmetry mismatch between the 10.5 bp per turn of DNA and the 12 bp translocation step (6 times 2) per full cycle. In the movie, translocation is first shown from the top (looking towards the α-subdomains), where the slow rotation of dsDNA due the DNA-hexamer base pair mismatch can be seen. In the following side view, one and later on five subunit(s) is (are) hidden for clarity. Subunit colours depict conformations throughout and correspond to Figure 4. Atomic motions are interpolations (morphs) between the six conformations observed in the translocating state III.E.

Movie S3. A more artistic presentation of the mechanism of dsDNA translocation by FtsKαβ. First, the FtsK hexamer is shown translocating DNA at various relative speeds. Then, the six conformational states are represented by a colour gradient and are shown to rotate around the ring, viewed from an upstream, and then downstream camera position. The concerted conformational changes in all six subunits are highlighted in the next scene where the six protomers are represented side-by-side, aligned by their α domains as in the top panel of Figure 2*A*. Then, similar to Movie S2, interactions of FtsK with DNA are highlighted using a side view in which two subunits are removed for clarity. The interacting basic residues are shown in red, the non-interacting ones in black and the FtsK footprint on the DNA backbone is coloured in yellow. Finally, the FtsK homo-hexamer is hidden to highlight that FtsK is in fact treadmilling on the backbone of the DNA. The movie has been produced by Phospho Biomedical Animation guided by instructions from the authors.
